# Chronic endometritis modifies decidualization in human endometrial stromal cells

**DOI:** 10.1186/s12958-017-0233-x

**Published:** 2017-03-04

**Authors:** Di Wu, Fuminori Kimura, Luyi Zheng, Mitsuaki Ishida, Yoko Niwa, Kimiko Hirata, Akie Takebayashi, Akiko Takashima, Kentaro Takahashi, Ryoji Kushima, Guangmei Zhang, Takashi Murakami

**Affiliations:** 1 Department of Obstetrics and Gynecology, Shiga University of Medical Science, Seta Tsukinowa-cho, Otsu, 520-2192 Japan; 2 Department of Obstetrics and Gynecology, 1st Affiliated Hospital, Harbin Medical University, Harbin, Heilongjiang Province 150001 China; 3 Department of Clinical Sciences and Laboratory Medicine, Kansai Medical University, 2-5-1 Shin-machi, Hirakata City, Osaka 573-1010 Japan; 4 Department of Clinical Laboratory Medicine, Shiga University of Medical Science, Seta Tsukinowa-cho, Otsu, 520-2192 Japan

**Keywords:** Chronic endometritis, Decidualization, IVF, Endometrial stromal cells, Sex hormone receptor

## Abstract

**Background:**

Chronic endometritis (CE) is a continuous inflammation of uterine endometrium, and it is usually symptomless. As CE has been thought not to affect the reproductive status and general health of affected women, its significance has not been explored. However, recent studies have shown that CE is related with repeated implantation failures after in vitro fertilization-embryo transfer, unexplained infertility, and recurrent miscarriages. As decidua differentiates to support the implantation process and maintains the pregnancy, we hypothesized that CE may influence the process of decidualization.

**Methods:**

Seventeen patients were employed in the experiment involving culture of endometrial stromal cells (ESCs). After obtaining endometrial samples, ESCs were harvested and cultured for 13 days. The concentrations in culture media and the protein expressions in ESCs of prolactin (PRL) and insulin-like growth factor binding protein-1 (IGFBP-1), two well known decidualization markers used in a large number of in vitro models, were analyzed by ELISA and Western blotting, respectively, and the cell numbers were also counted. The mRNA levels of PRL and IGFBP-1 were tested by quantitative real time polymerase chain reaction (RT-PCR). Since sex hormone induce proliferation and differentiation to decidua via binding to the sex hormone receptors (ERα, ERβ, PRA, and PRB), their expression was assessed in another 17 patients’ paraffin-embedded endometrial tissue specimens by immunohistochemistry and semi-quantified by H-score.

**Results:**

Increased cell numbers and reduced secretion of PRL and IGFBP-1 were detected by ELISA in the ESCs of CE patients after culture for 13 days compared with non-CE patients. The decreased protein expression of IGFBP-1 in ESCs of CE patients was detected by Western blotting. The decreased expression of PRL mRNA and IGFBP-1 mRNA were detected by RT-PCR. Increased expressions of ERα, ERβ, PRA, and PRB were observed in the stromal cells of CE patients in comparison to non-CE patients, whereas increased expressions of ERα and ERβ were detected in the glandular cells of CE.

**Conclusion:**

Our data suggests that CE modifies decidualization of human ESC through untuning the function of sex steroid hormone receptor.

**Electronic supplementary material:**

The online version of this article (doi:10.1186/s12958-017-0233-x) contains supplementary material, which is available to authorized users.

## Background

Chronic endometritis (CE) is a disease of continuous and subtle inflammation of the uterine endometrium. It is also called subclinical endometritis because it is usually symptomless or presents only with subtle symptoms such as abnormal uterine bleeding, pelvic pain, dyspareunia, and leucorrhea [1]. CE was thought to be caused by a variety of infectious agents such as bacteria, viruses, and parasites, but the precise cause of CE remains unclear [2]. It is diagnosed by the presence of plasma cells, which are never present under normal conditions, within the endometrial stromal layer to show the existence of a continuous immune response to some substance(s) or agent(s) in the endometrium [1, 3].

Since CE has been thought not to affect the reproductive status and general health of affected women [1], its significance has not been explored. However, recent research has shown a higher prevalence of CE in infertile patients (range 2.8 to 46%) [4–8] and relationships with recurrent abortion and repeated implantation failure [4, 9, 10]. Furthermore, a new clinical study reported that CE decreases the success rate of in vitro fertilization (IVF) [11]. These results suggest that CE affects uterine receptivity for the embryo and impairs successful pregnancy.

Endometrial stromal cells (ESCs) undergo the process of decidualization [12]. Decidua is transformed under the influence of estrogen and progestogen and differentiates to maintain the pregnancy. It exchanges nutrition, gas, and waste between the fetus and mother in cooperation with the placenta. Decidua also produces hormones, growth factors, and cytokines such as prolactin (PRL) [12–14], corticotrophin releasing factor (CRF) [15, 16], insulin-like growth factor binding protein-1 (IGFBP-1) [12, 17], and interleukin-15 [18, 19]. It may regulate the invasion of the trophoblast and the attack from the maternal immune system [20, 21]. Given the adverse effects of CE on the implantation process in clinical studies and the role of decidua, CE may modify the process of decidualization.

Another gynecological disease that may cause infertility and implantation failure is endometriosis, affecting 10–15% women of reproductive age [22, 23]. It is characterized by the appearance of hormone-sensitive endometrial-like tissue outside the uterine cavity [24]. Eutopic ESCs from endometriosis patients exhibit a reduced response to decidualization in primary culture, which suggests that abnormal differentiation of the eutopic endometrium is related to infertility [25, 26]. Recently, an article reported that 52.94% of endometriosis patients are diagnosed with CE compared to only 27.02% of non-endometriosis patients [27]. This fact also suggests that CE may be involved in the modification of decidualization in eutopic endometrium.

To explore the effects of CE on decidualization, the expressions of PRL and IGFBP-1, markers of decidualization, were analyzed in primary culture of human ESCs. In addition, the expressions of sex steroid hormone receptors, estrogen receptors α and β (ERα, ERβ) and progesterone receptors A and B (PRA, PRB) were analyzed by immunohistochemistry.

## Methods

### Collection of samples for culture experiment

The patients underwent urine LH testing provided from our hospital daily from 3 or 4 days before the estimated ovulation date. On the day or the day after the urine test became positive, the patients visited our hospital and the status of follicle growth was confirmed. When follicle growth was sufficient as preovulatory follicle, the patient was planned to undergo hysteroscopy and curettage at 7 or 8 days after predicted ovulation. When endometrial polyps were found at hysteroscopy, the patients were excluded. On the day of hysteroscopy and curettage, blood samples were obtained to measure serum levels of estradiol (E2) and progesterone (P4). Endometrial tissue obtained by first curettage from each patient was sent to the pathology department. Mild curettage was performed a few more times to obtain endometrial tissue for the culture experiment. The tissues of first curettage were fixed in 10% formaldehyde, embedded in paraffin, and sectioned at 4 μm. According to the criteria of Noyes et al., the section was stained with hematoxylin and eosin for histological examination and judging of the date of the endometrial phase [28]. Moreover, the sections were immunostained with anti-CD138 antibody (product no.B-A38, Nichirei Corp.) as we previously reported or used for immunostaining for sex steroid hormone receptors. The criteria of CE diagnosis was the observation under microscopy with a high powered field of two or more CD138-positive cells in the stromal layer [29]. The diagnosis of CE and endometrial dating was performed by investigators (FK, MI) and the investigators performing the culture experiments (DW, LZ, YN) were blinded to the CE status of the sample until all procedures of cell culture, cell counting and ELISA were completed and all results were obtained.

The patients’ age, parity, gravidity, body mass index (BMI), endometrial thickness, and causes of fertility including the diagnosis of endometriosis were obtained from the patients’ records. Endometriosis was diagnosed when the existence of endometrioma was confirmed by MRI or stage III or IV endometriosis was confirmed by laparoscopy within 2 years before participation in this study. The information with endometriosis was used for the subanalysis of following experiment as endometriosis may affect the result.

### Isolation and culture of ESCs

The endometrial tissues obtained from 17 patients, nine CE patients including five endometriosis patients and 8 non-CE patients including 4 endometriosis patients, were used to perform primary cell culture as we previously reported [30]. Briefly, after mince, these endometrial tissue pieces were incubated for 1 h at 37 °C in a humidified atmosphere of 5% CO_2_ in air with 0.2% collagenase (Sigma) and 0.005% deoxy ribonuclease I (Worthington Biochemical Co.), with gentle pipetting every 15 min. After digestion, 20 mL of DMEM/F12 (1:1) (1×) (Life technologies) replenished with 10% charcoal striped FBS (HyClone GE Healthcare Life Sciences) were added, and the cell suspension was placed in the upright position for 3 min. Then, the supernatant including the rich fraction of stromal cells was filtered through the 40-μm cell strainer (Falcon Life Sciences) and then centrifuged for 10 min at room temperature. After the pellet was resuspended, a total of 400,000 viable cells per well were transferred into six well multidish (Thermo Fisher Scientific). The ESCs were cultured at 37 °C in a humidified atmosphere of 5% CO_2_ in air with 2 mL of DMEM replenished with 10% FBS, 100 IU/mL penicillin, and 100 μg/mL streptomycin. After 1 h, the culture media were exchanged to culture media including estradiol 3 × 10^−10^ mol/L (81.7 pg/mL) (Sigma) and progesterone 5 × 10^−8^ mol/L (15.7 ng/mL) (Nacalai Tesque, Inc.). The culture media were changed every other day, and the ESCs were cultured for 13 days. The culture media were changed 24 h before the collection of culture media and cell harvest. The culture media were collected, centrifuged at room temperature followed by the collection of supernatant, and stored at −80 °C for later analysis. The numbers of ESCs in a dish were counted by the inverted microscope with cell counter after exposure to 1 mL of 0.05% trypsin and 0.02% EDTA in PBS for a few minutes, followed by deactivation by adding 1 mL of DMEM/F12 with 10% FBS.

### ELISA to detect PRL and IGFBP-1 in culture media

The levels of PRL and IGFBP-1 in culture media were measured by ELISA kits (Prolactin Picokine ELISA Kit, Product no. EK0593, Boster Biological Technology. Ltd.; Human Free IGFBP-1 Quantikine ELISA Kit, Product no. DGB100, RSD R&D Systems Inc.) following the manufacturers’ instructions. The concentrations of PRL secreted by a cell was calculated by the formula: [PRL] x [volume ratio]/cell number. Where [PRL] represents the concentration of PRL in culture media assessed by ELISA (pg/L), and [volume ratio] indicates the actual volume of collected medium in one well (mL) divided by 2 mL (the initial volume of culture media). Cell number means the total cell number in the well. The concentration of IGFBP-1 secreted by a cell was obtained using an identical calculation.

### Protein extraction from cultured cells and western blotting

After discarding the supernatant, the cells on wells were washed twice with PBS. RIPA buffer (200 μL: 20 μL 1% SDS, 20 μL RIPA Buffer with protease inhibitor cocktail and 160 μL DW, Nacalai Tesque, Inc.) was added to the well. The cells on wells were cooled for 5 min on ice, collected with the cell scraper to be homogenized, and centrifuged at 10000 × g at 4 °C for 10 min. The supernatant was collected and kept at −80 °C until used for Western blotting.

The protein (10 μg) was boiled in Laemmli solution under reducing condition at 95 °C for 7 min. The solutions from CE and non-CE patients and Protein Ladder One Triple-color (Nacalai Tesque) were electrophoresed on a 15% SDS–polyacrylamide gel (Wako Pure Chemical Industries) and then transferred to a polyvinylidene difluoride membrane (Millipore). Membranes were incubated with 20% blocking reagent-N102 (NOF Co.) in 25 mM TBS containing 0.1% Tween-20 (TBST; pH 7.4) at room temperature for 1 h to block non-specific protein binding sites, followed by incubation with the IGFBP-1 antibody (1: 1000; Santa Cruz Biotechnology, Inc.) or GAPDH (1:5000; Enzo Life Science, Inc.) diluted by TBST at 4 °C overnight. After washing with TBST for 20 min thrice, the membranes were reacted with a peroxidase-labeled anti-mouse IgG (1:50,000; GE Healthcare) for 1 h at room temperature. Peroxidase labeling was detected by chemiluminescence using the Chemi-Lumi one super (Nacalai Tesque).

### RNA extraction from culture cells and real-time polymerase chain reaction

Total RNA was extracted from ESCs after culture for 13 days with RNeasy Micro kit (QIAGEN.) following the manufacturer’s instructions. Each sample (100 ng) was reverse transcribed using the Prime Script RT Master Mix (Takara Biotechnology. Co., Ltd.). Real-time PCR was performed using the LightCycler 480 SYBR Green I Master (Roche Diagnostics International Ltd.) with specific primer pairs for PRL, IGFBP-1 and GAPDH as follows: PRL, F:5’- GCCCCCTTGCCCATCTGTCG-3’ and R:5’- AGAAGCCGTTTGGTTTGCTCC -3’; IGFBP-1, F:5’- TGCTGCAGAGGCAGGGAGCCC -3’ and R:5’- AGGGATCCTCTTCCCATTCCA -3’ (Takara); GAPDH, F:5’- AAATCCCATCACCATCTTCCA -3’ and R:5’- AATGAGCCCCAGCCTTCTC -3’ (Sigma).

The mixture of reaction reagents was incubated at 95 °C for 5 min and cycled according to the following parameters: 95 °C for 10 s, 55 °C for 15 s, and 72 °C for 10 s for a total of 45 cycles, followed by cooling for 10 s at 50 °C by LightCycler 480TL system II (Roche Diagnostics International Ltd.). Melting curve analysis was performed.

### Immunohistochemistry for endometrial tissue specimens

The stocked paraffin-embedded endometrial tissue specimens obtained from 156 infertile patients from 7 to 8 days after ovulation to diagnose the endometrial dating and CE were the candidates for the study of immunohistochemistry. The diagnosis of CE was done by the same method of the culture experiment of the present study. If endometrial disease such as endometrial hyperplasia or polyp were diagnosed, the samples were excluded from the study. Seventeen samples (eight samples of CE and nine samples of non-CE) were randomly chosen for the analysis by an investigator (FK) when the endometrial dating of the phase in the endometrium pathologically determined according to the criteria of Noyes et al. was in the phase between 7 and 9 days in the secretory phase [28]. At that time the investigator was blinded to the patients’ character except the diagnosis of CE and endometrial dating. The patients’ age, parity, gravidity, BMI, endometrial thickness, and causes of fertility were obtained from the patients’ records after the following experiments of immunohistochemistry were completely diagnosed by other investigators.

The paraffin-embedded blocks were cut into 4-μm-thick sections. After deparaffinization and rehydration, the slides were immersed into 3% Tritonx-100 in PBS for 15 min and heated in a pre-heated sodium citrate buffer (pH 6.0) at 98 °C for 20 min. After infusion in 3% hydrogen peroxide for 10 min, the sections were covered with 5% BSA in PBS for 25 min followed by incubation with the primary antibodies as follows: ERα (1:50; Product no. sc-8005, Santa Cruz Biotechnology) at 4 °C for 3 days, and ERβ (1:200; Product no. ab288, Abcam), PRA (use directly;Product no. PA0312, Leica Biosystems Newcastle Ltd.) and PRB (1: 200; Product no.3157S, Cell Signaling Technology, Inc.) at 4 °C overnight, incubated with horseradish peroxidase-conjugated secondary antibody for 30 min at room temperature, and developed with DAB. The slides were immersed into hematoxylin counterstain for 1 min, underwent gradient alcohol dehydration followed by the xylene process, and mounted with neutral resin for later observation under an inverted microscope (BH-2, Olympus Co.).

### Scoring of immunoreactivity for endometrial tissue specimens

In order to semi-quantitatively analyze the immunoreactivity of nuclear steroid receptors, the H-score was used [31]. Briefly, the cells of more than 500 ESCs or glandular cells were each scored using three degrees: strongly stained nuclei (two points), moderately stained nuclei (one point), and negative stained nuclei (zero points). The H-score was subsequently calculated by summing the numbers of each point multiplied by the percentage of the point.

### Statistical analysis

All data are expressed as means ± standard error of the mean (S.E.M). An unpaired *t*-test unpaired or the Mann Whitney test was performed based on whether that distribution was Gaussian using GraphPad Prism ver.7.00. A value of *P* < 0.05 was considered significant.

## Results

### CE affects the decidualization of ESCs

This study included samples of nine CE patients and eight non-CE patients (Table 1). Age, parity, gravidity, BMI, the levels of E_2_ and P_4_, endometrial thickness, and the rate of out-of-phase endometrium were not different between the CE and non-CE groups.

**Table 1:** Clinical characteristics of the patients participating in the study for ELISA, Western blotting and quantitative RT-PCR

	non-CE (*N* = 8)	CE (*N* = 9)	*P* value
Age (y)	37.9 ± 0.67	35.4 ± 1.80	0.25
Parity	1.00 ± 0.33	0.33 ± 0.24	0.11
Gravidity	0.38 ± 0.19	0.11 ± 0.11	0.23
BMI (kg/m^2^)	22.9 ± 1.32	22.9 ± 1.59	1.00
E2 (pg/mL)	117 ± 25.0	142 ± 29.14	0.54
P4 (ng/mL)	9.76 ± 1.86	13.21 ± 1.84	0.21
Endometrial thickness (mm)	8.89 ± 1.03	8.90 ± 0.65	0.99
Out of phase (%)	0.38 (3/8)	(0.67) 6/9	0.35
Cause of infertility	Male : 3	Male : 2	
	Tubal factor : 2	Tubal factor : 1	
	Endometriosis : 4	Endometriosis : 5	
	Unknown: 1	Unknown: 3	

Age, parity, gravidity, body mass index (BMI), the levels of E2 and P4, endometrial thickness, and the rate of out-of-phase endometrium were not different between the CE and non-CE groups

**Fig. 1:**
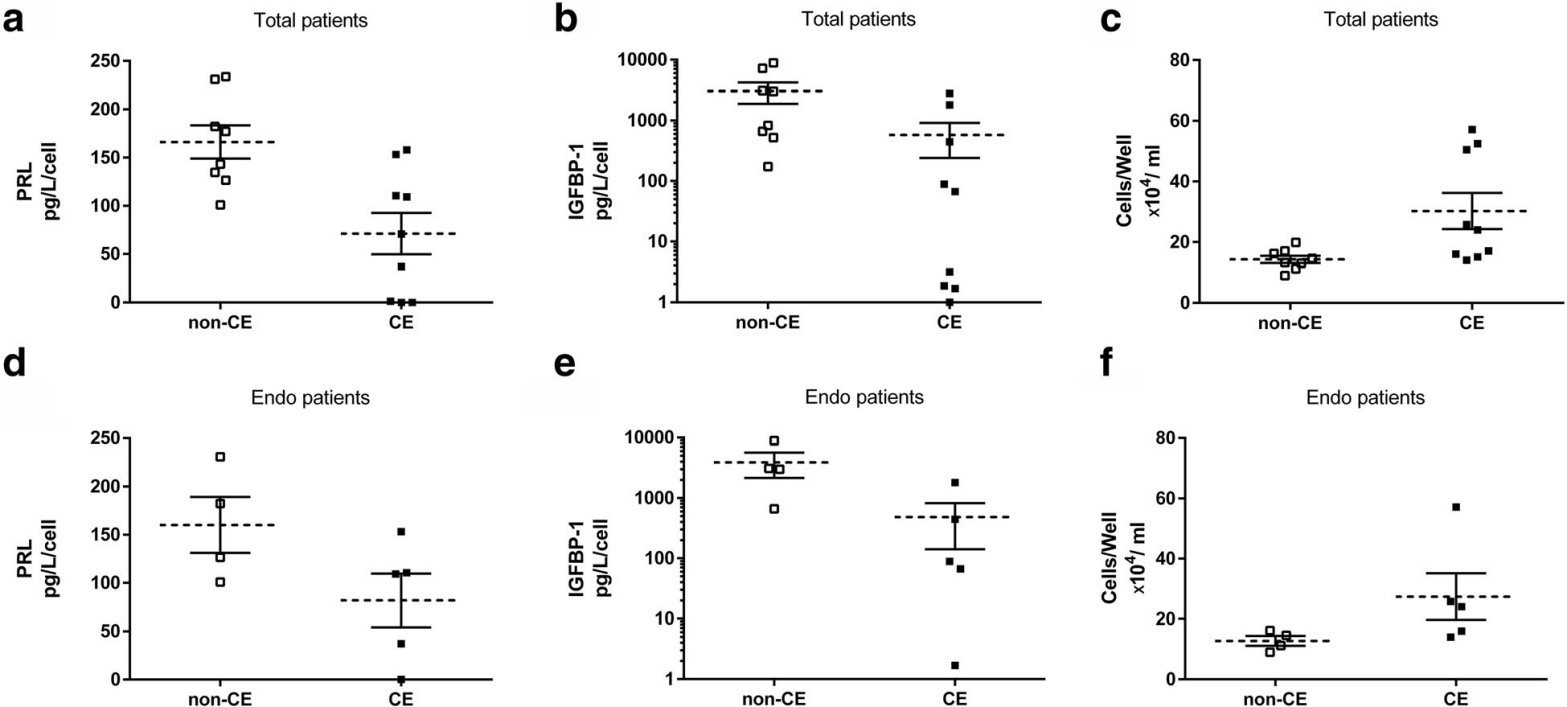
Secretion of PRL and IGFBP-1 from decidualized ESCs in vitro and cell numbers. Samples with endometriosis in the CE group and non-CE group were indicated by “Endo”. **a** The concentration of PRL in the media of cultured ESCs evaluated by ELISA. PRL concentration is lower in CE samples than in non-CE samples (*P* < 0.01). **b** The concentration of IGFBP-1 analyzed by ELISA. The data are shown using a log scale. Lower expression of IGFBP-1 is observed in CE samples than in non-CE samples (*P* < 0.01). **c** The number of stromal cells after culture for 13 days. It is obviously higher in the CE group than in the non-CE group (*P* < 0.05). **d** The concentration of PRL in the patients with endometriosis divided into the CE and non-CE groups. The level of PRL tends to be lower in the CE group than in the non-CE group (*P* = 0.09). **e** The concentration of IGFBP-1 in the patients with endometriosis separated into the CE and non-CE groups. The concentration of IGFBP-1 is lower in the CE group than in the non-CE group (*P* < 0.05). **f** The cell number of the patients with endometriosis divided into the CE and non-CE groups. The cell numbers are not different between the CE group and the non-CE group (*P* = 0.11)

The concentration of PRL secreted by a cell was obviously decreased in the CE group compared with the non-CE group (*P* < 0.01) (Fig. 1a). In addition, the concentration of another marker of decidualization, IGFBP-1, was also significantly reduced in the CE group compared with the non-CE group (*P* < 0.01) (Fig. 1b). The cell number of decidualized ESCs after culture for 13 days was obviously increased in the CE group compared with the non-CE group (*P* < 0.05) (Fig. 1c).

Since decidualization has been reported to be disturbed in the eutopic endometrium of endometriosis, only the samples with endometriosis were evaluated in the CE group (five patients) and non-CE group (four patients). In the patients suffering from both endometriosis and CE, the concentration of PRL tended to be decreased compared with the patients with endometriosis without CE (*P* = 0.09) (Fig. 1d). The concentration of IGFBP-1 produced by cells in patients with both CE and endometriosis was decreased compared with the endometriosis patients without CE (*P* < 0.05) (Fig. 1e). The cell numbers of patients with endometriosis was not increased in the CE group compared with the non-CE group (*P* = 0.11) (Fig. 1f).

**Fig. 2:**
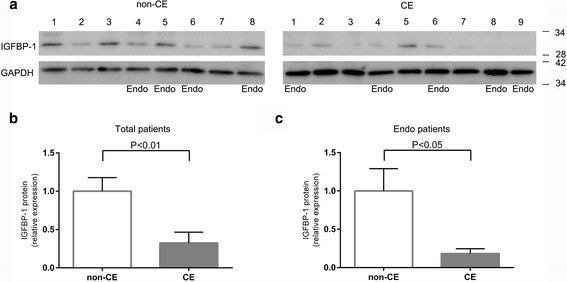
Western blotting of IGFBP-1 in ESCs. Samples with endometriosis in the CE group and non-CE group were indicated by “Endo”. **a** Western blotting of IGFBP-1 and GAPDH. Endo under the blots indicates endometriosis patient. **b** The ratio of production of IGFBP-1 per GAPDH in all patients. The production of IGFBP-1 is significantly reduced in the CE group compared to the non-CE group (*P* < 0.01). **c** The ratio of production of IGFBP-1 per GAPDH in endometriosis patients. The production of IGFBP-1 in patients with both CE and endometriosis is also decreased compared with endometriosis patients without CE (*P* < 0.05)

In addition, a different expression of IGFBP-1 in ESCs between CE and non-CE group was detected by Western blotting (*P* < 0.01) (Fig. 2a and b). A difference in the production of IGFBP-1 was also confirmed in patients with both CE and endometriosis compared with the endometriosis patients without CE by western blotting (*P* < 0.05) (Fig. 2a and c).

**Fig. 3:**
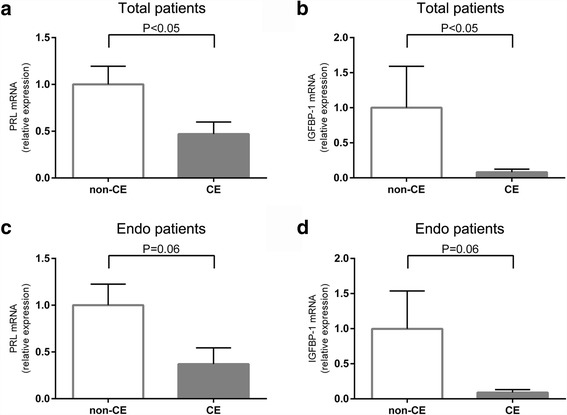
The mRNA levels of PRL and IGFBP-1 in decidualized ESCs in vitro. Samples with endometriosis in the CE group and non-CE group were indicated by “Endo”. **a** The mRNA level of PRL in the decidualized ESCs. The level is lower in the CE group. **b** The mRNA level of IGFBP-1 in the decidualized ESCs. The level is lower in the CE group. Patients with endometriosis are divided into the CE and non-CE groups. **c** and **d** the mRNA level of PRL and IGFBP-1tends to be lower in the CE group than in the non-CE group (*P* = 0.06 in both)

The mRNA levels of PRL and IGFBP-1 were significantly lower in the CE group than in the non-CE group (*P* < 0.05) (Fig. 3a and b). When only the samples with endometriosis were evaluated in the CE group (five patients) and the non-CE group (four patients), the mRNA levels of PRL and IGFBP-1 tended to be decreased in the CE group (*P* = 0.06 in both) (Fig. 3c and d).

### Immunohistochemistry of ERα, ERβ, PRA, and PRB

**Fig. 4:**
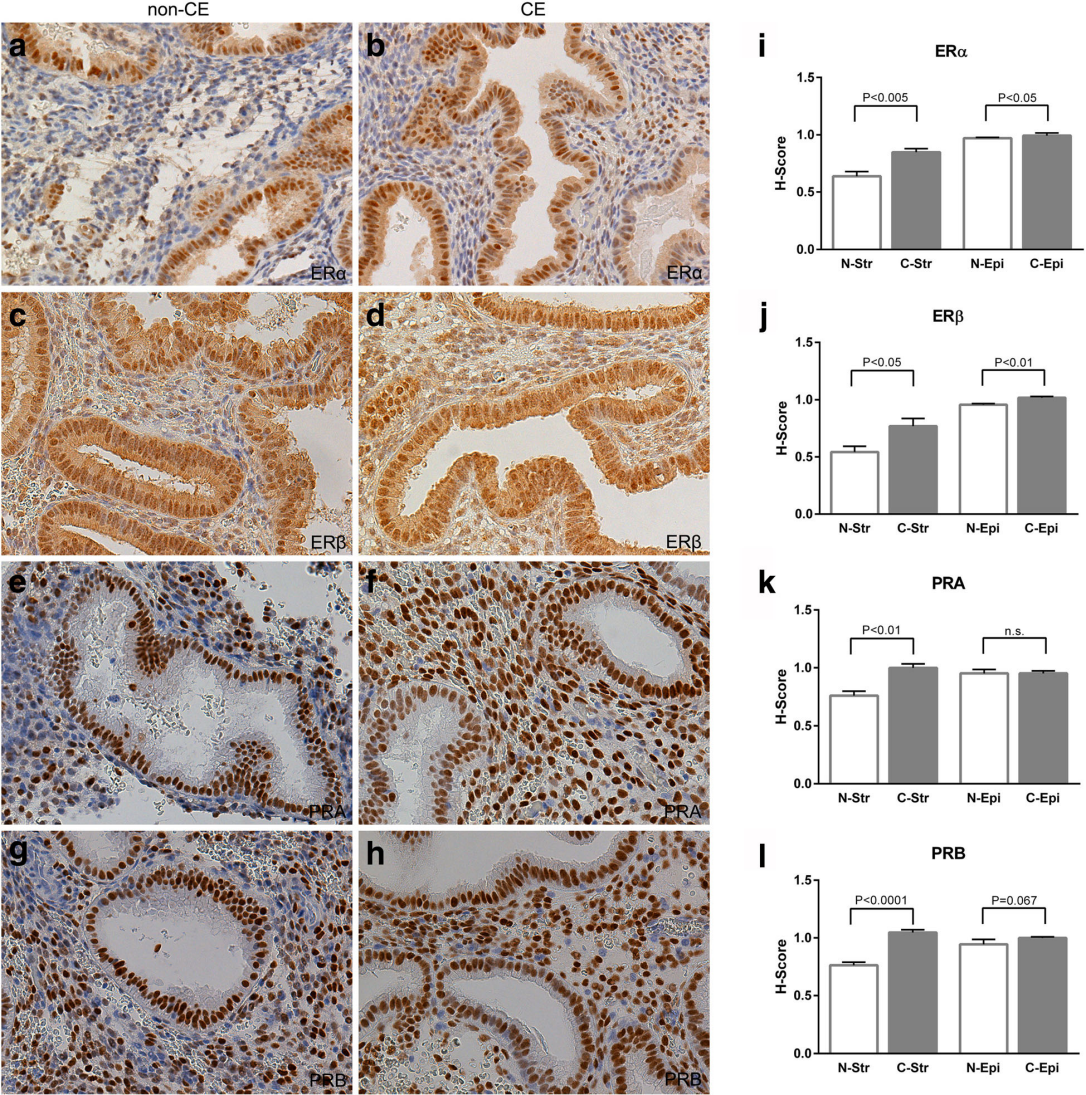
IHC staining for ERα, ERβ, PRA, and PRB in non-CE and CE patients. **a** ERα immunoreactivity in the non-CE group. **b** ERα immunoreactivity in the CE group. **c** ERβ staining in the non-CE group. **d** ERβ immunoreactivity in the CE group. **e** PRA immunoreactivity in the non-CE group. **f** PRA staining in the CE group. **g** PRB immunoreactivity in the non-CE group. **h**, PRB immunoreactivity in the CE group. N-Str, C-Str, N-Epi, and C-Epi mean stromal cells in the non-CE group, stromal cells in the CE group, epithelial cells in the non-CE group, and epithelial cells in the CE group, respectively. **i** The H-Score for ERα. The H-Score shows that ERα in stromal cells is higher in the CE group than in the non-CE group (*P* < 0.005), and the expression is higher in glandular cells of the CE group than of the non-CE group (*P* < 0.05). **j** The H-Score for ERβ. The H-Score shows that ERβ in stromal cells in the CE group is higher than in the non-CE group (*P* < 0.05), and the expression is higher in glandular cells of the CE group than of the non-CE group (*P* < 0.01). **k** The H-Score for PRA. The H-Score demonstrates that PRA in stromal cells is higher in the CE group than in the non-CE group (*P* < 0.01), but the expression is not significantly different in glandular cells of the non-CE and CE groups. **l** The H-Score for PRB. The H-Score shows that PRB in stromal cells in the CE group is much higher than in the non-CE group (*P* < 0.0001), but its expression level is similar in glandular cells of the non-CE and CE groups

The tissues for immunohistochemistry assay were obtained from 17 patients, including nine non-CE patients and 8 CE patients. Age, parity, gravidity, BMI, the levels of E_2_and P_4_ and endometrial thickness were not different between the CE and non-CE groups (Table 2). ERα, ERβ, PRA, and PRB immunoreactivities were examined on paraffin sections in the non-CE group (Fig. 4a, c, e, and g) and the CE group (Fig. 4b, d, f, and h). The H-Scores of these receptors in stromal cells and glandular cells in the non-CE group and the CE group are shown in Fig. 3 i, j, k, and l. Higher expressions of ERα and ERβ were present in stromal cells in the CE group (*P* < 0.005 and *P* < 0.05) and there was a significant difference in both stromal cells and glandular cells (*P* < 0.05 and *P* < 0.01), although the difference of absolute value of ERα and ERβ expressions in glandular cells were inconspicuous in the CE group in comparison with the non-CE group (Fig. 4a, b, c, d, i, and j). Higher expressions of PRA and PRB in stromal cells were seen in the CE group (*P* < 0.001 and *P* < 0.0001), whereas PRA and PRB expressions were similar in the epithelia between the CE group and the non-CE group (Fig. 4e, f, g, h, k, and l).

**Table 2:** Clinical characteristics of the patients participating in the study for IHC for sex steroid hormone receptors

	non-CE (*N* = 9)	CE (*N* = 8)	*P* value
Age (y)	37.0 ± 1.19	37.3 ± 2.61	0.24
Parity	0.44 ± 0.24	0.75 ± 0.25	0.34
Gravidity	0.11 ± 0.11	0.38 ± 0.18	0.24
BMI (kg/m^2^)	21.2 ± 0.86	20.7 ± 0.86	0.73
E2 (pg/mL)	109 ± 16.9	151 ± 28.1	0.093
P4 (ng/mL)	14.6 ± 1.66	18.1 ± 3.21	0.32
Endometrial thickness (mm)	9.40 ± 1.14	8.67 ± 1.54	0.70

Age, parity, gravidity, body mass index (BMI), the levels of E2 and P4 and endometrial thickness were not different between the CE and non-CE groups

## Discussion

Although previous studies suggested that CE affected the uterine receptivity of the endometrium to embryo, little information is available about the mechanism. This is the first study to assess the effect of CE on decidualization of ESCs. Decidualization plays crucial roles in trophoblast invasion, the endometrial immune response of the feto-maternal interface [20, 21, 32], and the establishment of the placenta. These may suggest that disturbance of the decidualization process emerges the correlation with infertility and miscarriage [33, 34].

In the present study, the ESCs of CE had significantly lower expressions and secretion of PRL and IGFBP-1 in vitro after the induction of decidualization compared with the ESCs of non-CE. Moreover, the number of ESCs after culture for 13 days with E_2_ and P_4_ treatment to induce decidualization was significantly higher in the CE group than in the non-CE group. The modification of differentiation was connected with the promotion of proliferation. CE modifies decidualization in vitro and weakens the action of P_4_ on ESCs, resulting in less potential to differentiate and greater potential to proliferate. In 3 CE patients with no or extremely low levels of PRL and IGFBP-1 secretion, the number of ESCs was extremely high, approximately 3-fold higher than that in non-CE patients (Fig. 1), and the ESCs proliferated and differentiated like as exposed to only E_2_.

In the culture experiment, CE was diagnosed in nine of 17 patients, endometriosis was diagnosed in nine of 17 patients, and five of 17 patients was suffered from CE and endometriosis. The main reason why the samples we obtained included higher percentage of CE and endometriosis patients is thought to be that we chose patients of endometriosis and refractory infertility when we enrolled as these were the risk of CE.

Previous studies reported that the capacity of eutopic endometrial stromal cells for decidualization was reduced, whereas the proliferation potential was increased in endometriosis patients [25]. Since the present result might be due to the effect of endometriosis, only samples with endometriosis were chosen and analyzed. Our data shown that the significantly reduced protein level and mRNA expression of PRL and IGFBP-1 and the increase of cell number were oppositely observed in the CE patients compared with non-CE patients, no matter whether the patients have endometriosis or not. This result suggest that CE affected the differentiation of cultured ESCs and modified decidualization independently of endometriosis.

Although the number of samples in this experiment was relatively low, a significant difference was apparent between CE and non-CE in terms of the secretion of decidual proteins and cell numbers. This may cause by the specifically developed methods for this experiment. The first thing is that the endometrial samples were obtained in the mid-secretory phase. CE is thought to be caused by the continuous and subtle infection of some agent inside the uterus. When the ESCs obtained in the proliferative phase are used in primary culture, the induction of decidualization starts with the regulation of inflammation by antibiotics in the culture media. The removal of endometrial cells from the uterus with CE leads to the cessation of exposure to inflammation and immune cells in the uterus and the beginning of treatment for the infection with antibiotics in vitro. However, using the sample obtained in the secretory phase, the ESCs are already exposed to inflammation and immune cells during the initial process of decidualization in vivo. As reported in the present study, the expression of sex steroid receptors is already modified at the mid-secretory phase in in vivo endometrium of CE patients even with the collection of the endometrial date for the phase.

The second point is that lower concentrations of E_2_ and P_4_ were used in the present study compared with previous studies, whereas the concentrations are identical to in vivo conditions. In many previous reports higher levels of E_2_ and P_4_ than in the in vivo condition, cyclic adenosine monophosphate (cAMP) or medroxyprogesterone acetate (MPA) have often been used to in vitro experiments to make the experiments stable [35–37]. We previously reported that the difference in the response to secrete decidual proteins becomes larger among samples with a decreased P_4_ concentration in the medium [30]. These suggested that milder impairment of the endometrium by CE may be masked with exposure to higher levels of E_2_, P_4_, cAMP, or MPA.

Mishra et al. have already shown the higher expressions of ER and PR in ESCs in the secretory phase of CE patients and the result is identical to our result although the expression of hormone receptor subtypes was not investigated. The effects of estrogen develop in the endometrium through two main classical estrogen receptor (ER) isoforms, ERα and ERβ, whereas progesterone acts by binding its two predominantly progesterone receptor (PR) isoforms, PRA and PRB [38, 39]. ERα and PRA are the predominant isoforms that mediate the critical functions of the uterus for the implantation process and maintenance of pregnancy [40, 41]. PRA is the primary mediator during the secretory phase of decidualization in humans [42]. The expressions of ERα, PRA, and PRB decline gradually, both in glandular cells and stromal cells, during the mid-secretory phase. ERβ appears to show no change throughout the secretory phase of the menstrual cycle [42, 43]. Since there are subtypes of ER and PR and the expressions of sex steroid hormone receptors change with the menstrual cycle, the expressions of subtypes of ER and PR were examined only in endometrial samples of the mid-secretory phase with endometrial dating between day 7 and 9 after ovulation in the present study. The results showed that the immunoreactivities of ERα, ERβ, PRA, and PRB in stromal cells were higher in the CE group, and the expressions of ERα and ERβ were significantly higher in the glandular cells of the CE group. These results suggest that the expressions of ERs and PRs in stroma and ERs in epithelia are already different in the mid-secretory phase in CE patients, even though the endometria differentiate similarly in morphology. Furthermore, we could not detect protein expression for PRL and IGFBP-1 in the same mid secretary samples by IHC and Western blotting (data not shown). By using RT-PCR only very low or almost null levels of PRL and IGFBP-1 mRNAs were detected (Additional file 1: Figure S1). Thus, at the mid secretary phase, decidualization is still in its early phase and the secretion of decidualization markers cannot be detected. Taken together, the abnormal expression of sex hormone receptors results in difficulty for endometrium to initiate the decidualization process and modifies the development of the endometrium for decidualization.

Since CE has been reported to cause recurrent implantation failure after IVF and spontaneous abortion [9, 44], many researchers have attempted to determine why CE resulted in poor outcomes of the implantation process. A recent report showed that altered uterine contractility in CE patients leads to uterine dysperistalsis, which may be a significant cause of infertility [45]. In women with CE, an abnormal pattern of lymphocyte subsets and, consequently, an aberrant endometrial microenvironment have been demonstrated [46]. This may be another possibility explaining how CE affects the success of pregnancy. However, decidualization is a key process to achieve implantation and pregnancy. This is the first report showing that CE modifies the process of decidualization. Our results may provide fundamental evidences for understanding how CE modifies the decidualization process, consequently, influence the implantation success of IVF and spontaneous abortion.

## Conclusions

In summary, the present study showed that in vitro decidualization was modified in CE patients. Furthermore, CE had an effect on the process of decidualization in dependently of the effect of endometriosis. The sex steroid receptors are aberrantly expressed in the endometrium of CE patients. These findings suggest that CE affects uterine receptivity for the embryo and maintenance of the pregnancy by the modification of decidualization through aberrant tuning of sex steroid hormones and their receptors.

## Additional file

Additional file 1:
**Figure S1.** The mRNA levels of PRL (**a**) and IGFBP-1 (**b**) in endometrial samples detected by RT-PCR. Total RNA was extracted from six endometrial samples (three non-CE and three CE) obtained at mid secretary phase. The expression levels of PRL and IGFBP-1 mRNAs were very low or almost null and there was no significant difference between CE group and non-CE group. (TIF 88 kb)

## Abbreviations

BMI: Body mass index
cAMP: Cyclic adenosine monophosphate
CE: Chronic endometritis
E_2_: Estradiol
ER: Estrogen receptor
ERα: Estrogen receptor α
ERβ: Estrogen receptor β
ESC: Endometrium stromal cell
IGFBP-1: Insulin-like growth factor binding protein-1
IVF: In vitro fertilization
MPA: Medroxy progesterone acetate
P_4_: Progesterone
PR: Progesterone receptor
PRA: Progesterone receptor A
PRB: Progesterone receptor B
PRL: Prolactin
RT-PCR: Reverse transcription polymerase chain reaction
