# Supplementary figures and images for: Chronic endometritis modifies decidualization in human endometrial stromal cells
Source: Reprod Biol Endocrinol. 2017 Mar 4;15:16. doi: 10.1186/s12958-017-0233-x (PMC5336610; doi:10.1186/s12958-017-0233-x)

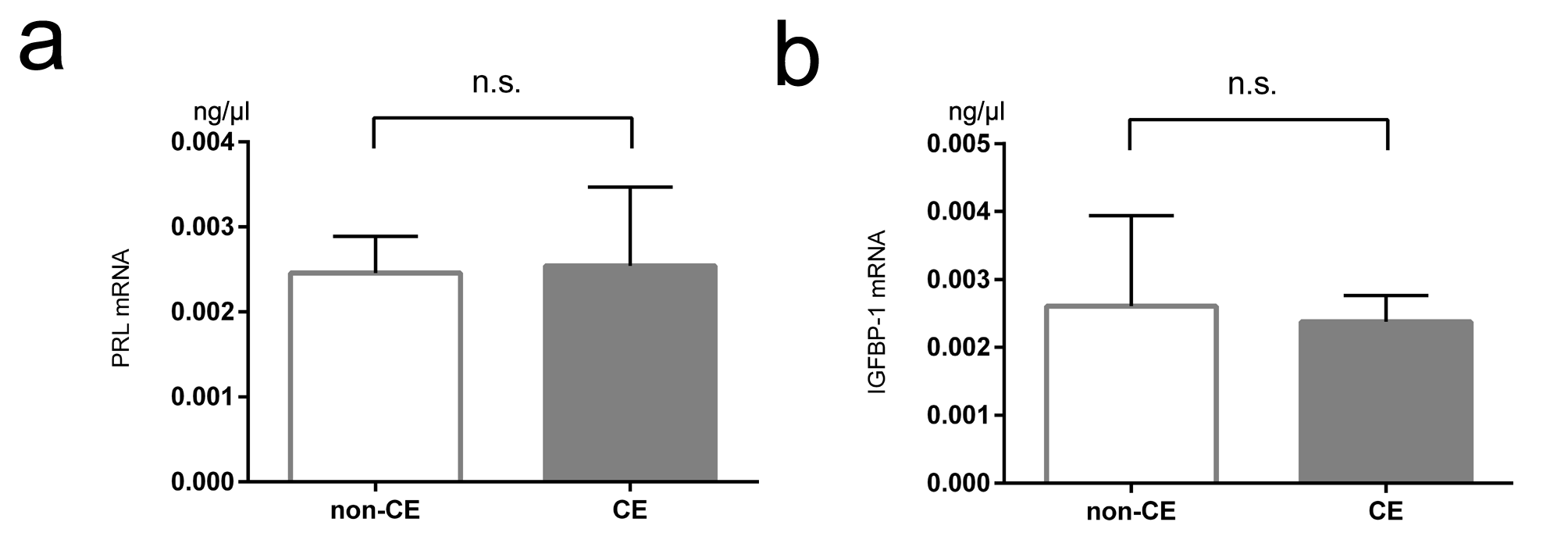

Supplement: Additional file 1: — Figure S1. The mRNA levels of PRL (a) and IGFBP-1 (b) in endometrial samples detected by RT-PCR. Total RNA was extracted from six endometrial samples (three non-CE and three CE) obtained at mid secretary phase. The expression levels of PRL and IGFBP-1 mRNAs were very low or almost null and there was no significant difference between CE group and non-CE group. (TIF 88 kb) [file 12958_2017_233_MOESM1_ESM.tif]
